# Supplementary figures and images for: Repurposed small molecule toxin inhibitors neutralise a diversity of venoms from the Neotropical viperid snake genus Bothrops
Source: eLife. 2026 Jul 31;15:RP110419. doi: 10.7554/eLife.110419 (PMC13427344; doi:10.7554/eLife.110419)

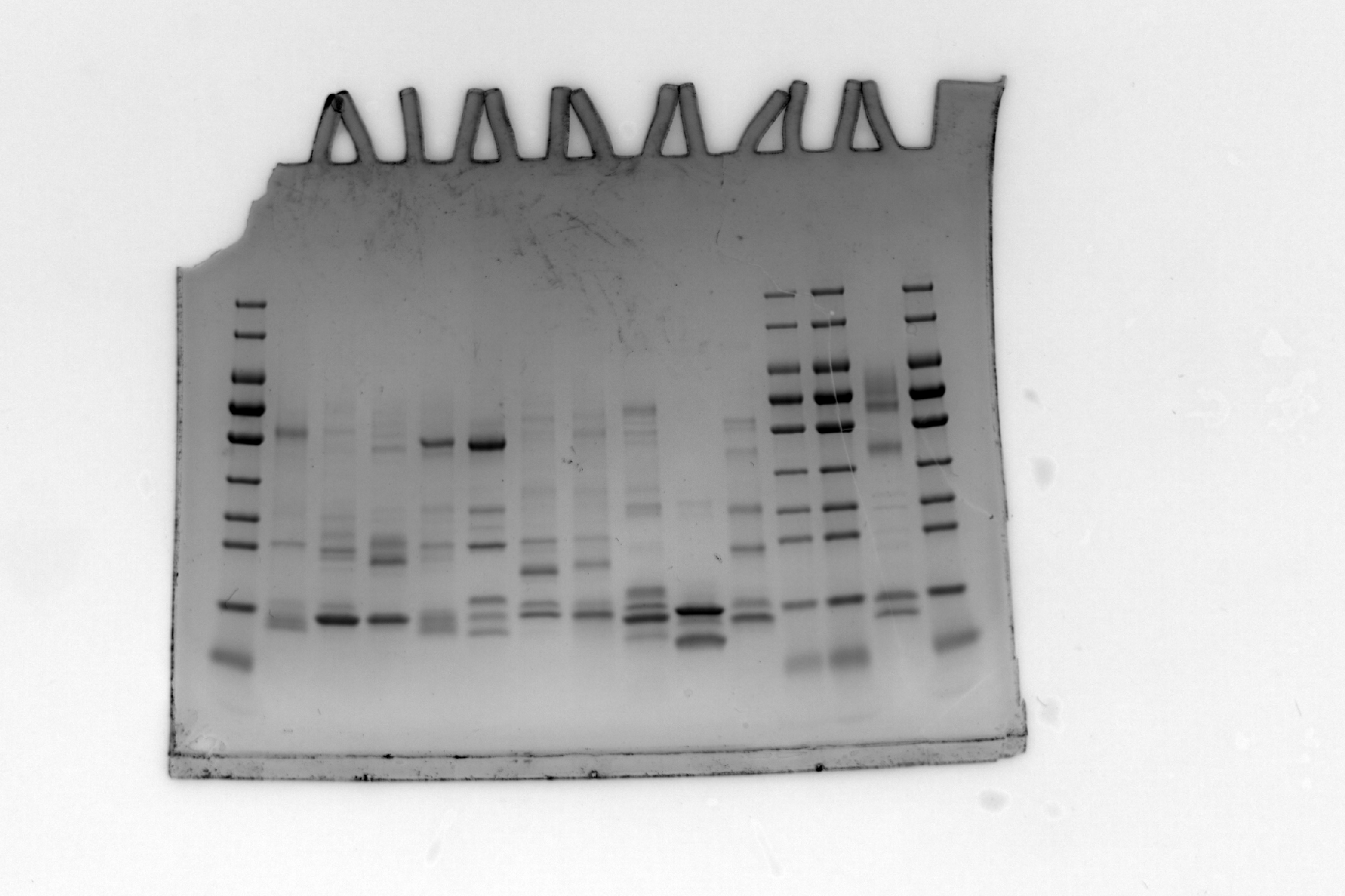

Supplement: Figure 2—source data 1. [file elife-110419-fig2-data1.zip › Figure 2A-source data 1/Figure 2-source data 1.Original SDS-PAGE gel displayed in figure 2A.tif]

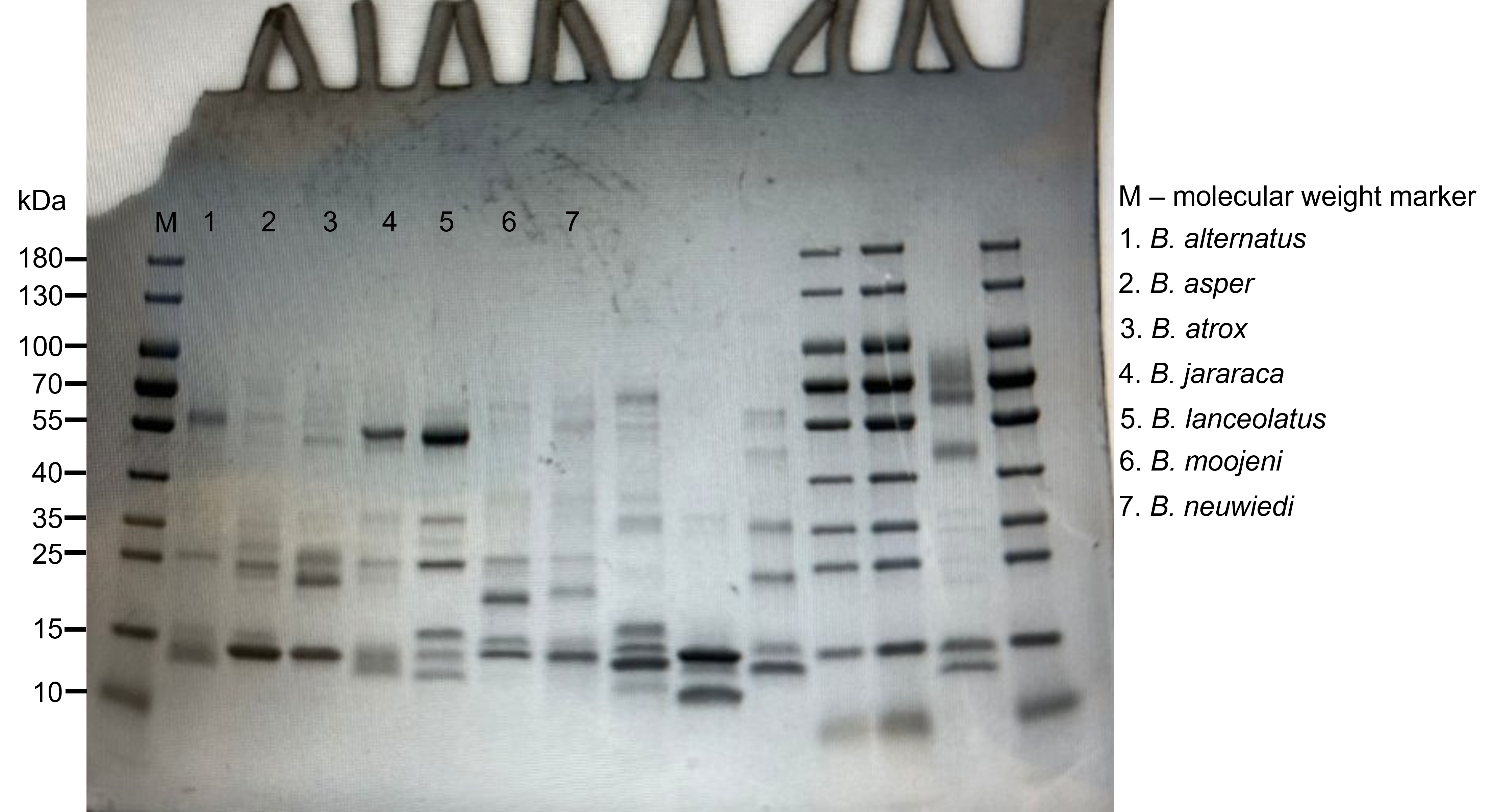

Supplement: Figure 2—source data 2. [file elife-110419-fig2-data2.zip › Figure 2A-source data 2/Figure 2-source data 2 - Original SDS-PAGE gel labelled with the 7 Bothrops profiled as displayed in figure 2A.tif]
