# Supplementary material for: Repurposed small molecule toxin inhibitors neutralise a diversity of venoms from the Neotropical viperid snake genus Bothrops
Source: eLife. 2026 Jul 31;15:RP110419. doi: 10.7554/eLife.110419 (PMC13427344; doi:10.7554/eLife.110419)
Supplement: Supplementary file 1. [file elife-110419-supp1.docx]

| **Supplementary Table 1. *Bothrops* Venom Compositions from published proteomics data** | | | | | |  |  |
| --- | --- | --- | --- | --- | --- | --- | --- |
|  |  | **% venom composition** | | |  |  |  |
| **Species name** | **Common name** | **PLA2** | **SVSP** | **SVMP** | **Captive bred or Wild caught location** | **Sampling information (individual or pool, gender, age)** | **Reference** |
| *Bothrops alternatus* | Urutu | 29.9 | 35.1 | 11.5 | captive bred - CEVAP/UNESP, SP, Brazil | Pool (numbers not defined) from adult specimens (gender not defined) | 1 |
|  |  | 2.0 | 5.8 | 52.2 | captive bred - Instituto Butantan (*Rhinocerophis alternatus*) | Pool of >10 adults of both sexes | 2 |
| *Bothrops asper* | Terciopelo | 28.8 | 18.2 | 41.0 | wild caught - Caribbean region of Costa Rica (San Carlos) | Pool of 15 adult specimens | 3 |
|  |  | 45.1 | 4.4 | 44.0 | wild caught - Pacific region of Costa Rica | Pool of 11 adult specimens |  |
|  |  | 6.2 | 4.4 | 30.9 | wild caught - 11 Ecuadorian provinces (min and max data presented) | 36 adult and 9 juveniles pooled by geographic location, lineage or ontogeny. Each pool ranged from 1 to 9 individuals. | 4 |
|  |  | 30.7 | 15.7 | 47.4 |  |  |  |
|  |  | 0.7 | 4.9 | 39.7 | wild caught - municipalities within the Department of Cauca in south-western Columbia (min and max data presented) | 10 adults pooled by geographic location, lineage or ontogeny. Each pool ranged from 2 to 4 individuals. | 4 |
|  |  | 23.0 | 9.3 | 46.6 |  |  |  |
| *Bothrops atrox* | Common lancehead | 5.7 | 9.7 | 46.5 | wild caught from Para region of Brazil (min and max data presented) | 37 adult specimens, male and female, with sizes ranging from 71.2 to 124.5 cm | 5 |
|  |  | 7.5 | 14.1 | 54.0 |  |  |  |
|  |  | 24.1 | 10.9 | 48.5 | captive bred | pooled samples from adult offspring of wild caught specimens | 6 |
|  |  | 14.3 | 4.6 | 72.1 | captive bred (Latoxan) | pooled adult specimens |  |
|  |  | 5.5 | 0.5 | 25.8 | wild caught from 14 locations in Venezuela and Brazil (min and max data presented) | Pools ranged from 1 to 40 specimens | 7 |
|  |  | 48.0 | 19.0 | 85.0 |  |  |  |
|  |  | 11.0 | 11.1 | 58.2 | wild caught from the rainforest region of Alto Marañon, Peru | pool of samples from 11 specimens (37-45 cm total length) | 8 |
|  |  | 3.3 | 8.1 | 54.6 | captive bred - Instituto Butantan | Pool of >10 adults of both sexes | 2 |
|  |  | 19.5 | 11.0 | 40.2 | captive bred or wild-caught from French Guiana, Peru, and Brazil(Latoxan - batch 211.191) | pool of samples from 76 snakes including males and females | 9 |
| *Bothrops jararaca* | Jararaca | 3.7 | 13.7 | 35.6 | wild caught from various locations in the southeast of Brazil | Pool of 20 specimens of adults and juveniles | 10 |
|  |  | 20.2 | 28.6 | 10.3 | wild caught from various locations in the south of Brazil | Pool of 13 specimens of adults and juveniles |  |
|  |  | 4.9 | 45.0 | 19.8 | captive bred - CEVAP/UNESP, SP, Brazil | Pool (numbers not defined) from adult specimens (gender unknown) | 1 |
|  |  | 3.2 | 11.7 | 42.8 | captive bred - Instituto Butantan (*Bothropoides jararaca*) | Pool of >10 adults of both sexes | 2 |
| *Bothrops lanceolatus* | Martinique lancehead | 8.6 | 14.4 | 74.2 | wild caught from Martinique 5-10 years prior to sampling (Latoxan) | Pooled from >12 specimens | 11 |
|  |  | 15.7 | 11.4 | 41.4 | wild-caught snakes from Martinique (Latoxan - batch 411.171) | Pool of two males and one female adult specimens | 9 |
| *Bothrops moojeni* | Brazilian lancehead | 39.3 | 13.4 | 21.9 | captive bred - CEVAP/UNESP, SP, Brazil | Pool (numbers not defined) from adult specimens (gender not defined) | 1 |
|  |  | 11.5 | 14.7 | 39.8 | Wild caught - in the region of Ribeirão Preto, SP, Brazil, | single male (pool of >3 extractions) | 12 |
|  |  | 17.1 | 19.8 | 36.5 |  | single female (pool of >3 extractions) |  |
| *Bothrops neuwiedi* | Jararaca pintada | 8.4 | 8.8 | 49.9 | captive bred - Instituto Butantan (*Bothropoides neuwiedi*) | Pool of >10 adults of both sexes | 2 |

*^1^Cavecci-Mendonca et al 2023, ^2^Sousa et al 2013, ^3^Alape-Girón et al 2009,  ^4^Mora-Obando et al. 2020, ^5^Sousa et al 2017, ^6^Núñez et al 2009,  ^7^Calvete et al 2011,  ^8^Kohlhoff et al 2012,  ^9^Larreche et al 2023,  ^10^Gonçalves-Machado et al 2016, ^11^Gutiérrez et al 2008, ^12^Amorim et al 2018.*
