# Supplementary material for: Repurposed small molecule toxin inhibitors neutralise a diversity of venoms from the Neotropical viperid snake genus Bothrops
Source: eLife. 2026 Jul 31;15:RP110419. doi: 10.7554/eLife.110419 (PMC13427344; doi:10.7554/eLife.110419)
Supplement: Peer review history

# Editor report: eLife Assessment

**DOI:** 10.7554/eLife.110419.3.sa0

**Authors:** Warren Andrew Andayi

**Affiliations:** Murang'a University of Technology Kenya

**Keywords:** Important, Solid

The findings of this study are **important** since they cover the repurposing of small molecules as metalloprotease and phospholipase inhibitors for early intervention in the treatment of bothropic envenoming in the Neotropics, and thus provide a strong rationale for the progression of these inhibitors into future preclinical and clinical evaluation for snakebite indications across various ecological zones, albeit the current evidence casts doubts on the viability of repurposing nafamostat. The strength of the evidence is **solid**; however, there are some weaknesses, such as a lack of translatability of the in vivo model and insufficient venom characterization. Thus, the strength of the evidence can be enhanced by using a mouse model in future studies. The paper remains of interest to ophiologists, biochemists and medicinal chemists.

# Referee report: Reviewer #1 (Public review)

**DOI:** 10.7554/eLife.110419.3.sa1

Very nice and coherent body of work with appropriate *in vitro* to *in vivo* transition in methods.

Lovely and easy to follow figures that can be understood even without the manuscript.

My recommendation is that a sentence or two be added clearly stating the authors think nafamostat is off the table and suggest other approaches/drugs that might be considered instead of just making a general statement. I think all this can be done in a few sentences.

Gabexate was administered to a snakebite victim in this case report from about 20 years ago and also a good example of the now better recognized threat to pregnancy.

Nasu K, Ueda T, Miyakawa I. Intrauterine fetal death caused by pit viper venom poisoning in early pregnancy. Gynecol Obstet Invest. 2004;57(2):114-6. doi: 10.1159/000075676. Epub 2003 Dec 19. PMID: 14691344

# Referee report: Reviewer #2 (Public review)

**DOI:** 10.7554/eLife.110419.3.sa2

Summary:

The authors set out to test whether a defined set of small molecules can lessen damaging effects caused by venoms from several Bothrops species, and whether these effects are consistent enough to suggest a broadly applicable approach. They present a cross-venom dataset spanning in-vitro activity readouts and blood-based functional outcomes, and include a chicken embryo model to explore whether venom inhibition can translate into improved survival. The central message is that certain small molecules can reduce specific venom-driven effects across multiple samples, providing a comparative resource for the field and a basis for prioritizing future validation.

Strengths:

The main value of this work is the breadth and structure of the dataset, which places multiple venoms and multiple readouts into a single, comparable framework that should be useful for readers evaluating patterns across samples. The experimental flow is generally coherent, moving from activity measurements to functional outcomes and then to an in-vivo test, which helps the reader understand how the authors link mechanism-oriented assays to more integrated endpoints. The manuscript also provides practical information for the community by highlighting which readouts appear most consistently affected across venoms, which can help guide hypothesis generation and study design in follow-up work.

Comments on revisions:

I would like to thank the authors for answering my questions. The manuscript has gained in quality, knowing the limitations that are now better stated in the manuscript.

# Author comment: Author response

**DOI:** 10.7554/eLife.110419.3.sa3

**Authors:** Rachel H Clare, Adam Westhorpe, Emma Stars, Taline D Kazandjian, Laura-Oana Albulescu, Stefanie K Menzies, Nicholas R Casewell

**Affiliations:** Edge Hill University Ormskirk United Kingdom; Liverpool School of Tropical Medicine Liverpool United Kingdom; Liverpool School of Tropical Medicine Liverpool United Kingdom; Liverpool School of Tropical Medicine Liverpool United Kingdom; Liverpool School of Tropical Medicine Liverpool United Kingdom; Lancaster University Lancaster United Kingdom; Liverpool School of Tropical Medicine Liverpool United Kingdom

The following is the authors’ response to the current reviews.

We thank the editors and reviewers for their assessment of this manuscript, and for the positive words highlighting the value of undertaking evaluation of small molecule drugs for snakebite in the neotropics, inclusive of the quality of this work and the value of the validated screening pipeline. We completely agree that the next steps for this work will be to evaluate the preclinical efficacy of the identified drugs in mouse models, though this considerable undertaking will form the basis of future work. Critically, the pipeline that we describe herein facilitates the selection of the most appropriate candidates to progress into such mouse studies, aligning with the 3Rs principles for minimising the need for animal research. The comment around insufficient venom characterisation seems somewhat misplaced – the objective of this project was not to characterise the venoms used, but to evaluate the *in vitro* inhibition of venom toxin family activities and identify the potential utility of specific repurposed drugs as therapeutics for snakebite in the neotropics. Venom characterisation of the diverse samples used in this project would represent an entire project and manuscript in its own right. We are pleased that the reviewers highlight the gap in research on serine protease inhibitors and the value this paper has in highlighting that more research is required in this area to identify a candidate that is more suitable for future clinical use than nafamostat.

The following is the authors’ response to the original reviews.

**Public Reviews:**

**Reviewer #1 (Public review):**

Summary:

Small molecule therapeutics for snakebite have received a lot of attention for their potential to close the gap between bite and treatment, where antivenom is not immediately available.

Strengths:

There has been a lot of focus on Africa, Asia, and India, but very little work related to neotropical regions. The authors seek to begin filling this gap in the preclinical literature. The authors use well-developed methods for preclinical assessment.

Weaknesses:

A clearer and more focused discussion of the limitations of the overall present work would be desirable (e.g. protection vs. rescue, why marimastat over prinomastat for in vivo assays when both have been through clinical trials for other indications; real-world feasibility of nafamostat, which has a half-life of 1-2 minutes compared to camostat, which has a half-life of hours). All of this could be improved in a revision.

We thank the reviewer for their shared opinion of the potential value of small molecules as snakebite envenoming therapeutics and their insight on the gap in focus in the neotropics, which this manuscript aims to address.

Our work in this manuscript included standard practice of pre-incubation between drug and venom for all *in vitro* studies, and sequential (i.e. not co-incubation) administration in the egg model. In our revised manuscript we will make these distinctions clearer. Use of a ‘rescue’ approach in the *in vitro* assays is not feasible due to the rapid destruction of the substrates used for assay readouts. The clearest rationale for the use of rescue models relates to their power within *in vivo* preclinical models (i.e. murine envenoming models) which, following the *in vitro* characterisations presented in this paper, are the logical next step for evaluating small molecule drugs for inhibiting neotropical snake venoms.

Although both marimastat and prinomastat are repurposed drugs that have undergone clinical evaluation for other indications, marimastat has been more extensively characterised preclinically than prinomastat for snakebite, and will soon enter Phase II clinical trial evaluation for this indication (https://www.ddw-online.com/ophirex-to-produce-snake-venom-inhibitor-for-lstm-study-40669-202602/). Marimastat also has a longer half-life in humans of 8-10 hours (Millar et al. 1998), compared to prinomastat (2-5h, Hande et al. 2004). We will more clearly highlight the rationale for selecting marimastat in the revised manuscript.

Although we appreciate the reviewer’s point regarding the short half-life of nafamostat (which is typically given by continuous iv infusion due to its short half-life), in the manuscript we have already stated that we do not recommend the progression of nafamostat as a snake venom serine protease (SVSP) inhibitor candidate due its low efficacy and off target effects. We highlight the need for the community to identify other serine protease inhibitors that might have utility for snakebite.

**Reviewer #2 (Public review):**

Summary:

The authors set out to test whether a defined set of small molecules can lessen damaging effects caused by venoms from several Bothrops species, and whether these effects are consistent enough to suggest a broadly applicable approach. They present a cross-venom dataset spanning in-vitro activity readouts and blood-based functional outcomes, and include a chicken embryo model to explore whether venom inhibition can translate into improved survival. The central message is that certain small molecules can reduce specific venom-driven effects across multiple samples, providing a comparative resource for the field and a basis for prioritizing future validation.

Strengths:

The main value of this work is the breadth and structure of the dataset, which places multiple venoms and multiple readouts into a single, comparable framework that should be useful for readers evaluating patterns across samples. The experimental flow is generally coherent, moving from activity measurements to functional outcomes and then to an in-vivo test, which helps the reader understand how the authors link mechanism-oriented assays to more integrated endpoints. The manuscript also provides practical information for the community by highlighting which readouts appear most consistently affected across venoms, which can help guide hypothesis generation and study design in follow-up work.

Weaknesses:

Several aspects of the study design and framing reduce the confidence with which readers can translate the findings beyond the specific experimental context presented. The evidence base is strongest in controlled in-vitro settings, while the bridge to real-world effectiveness remains limited, particularly for understanding performance under conditions that better reflect delayed treatment and systemic exposure. As a result, the manuscript is best interpreted as a well-organized comparative screening study with promising signals, rather than a definitive demonstration of a broadly effective, deployable intervention.

We appreciate the reviewer’s opinion on the thorough and logical workflow we present in this manuscript and the value this pipeline providers the field for future and parallel work. We agree with the reviewer that this provides a well-organized comparative screening study applicable to different snake species or therapeutics. In relation to the comment on this manuscript being a definitive demonstration of a broadly effective, deployable intervention we agree with their opinion and are happy to clarify that while the evidence presented in this manuscript is promising, there is much work still to do before such molecules are ready for deployment for treating snakebite. Ultimately, this manuscript supports the growing evidence of the promising utility of marimastat and varespladib, and extends this evidence to neotropical snake venoms in a comparative manner. The next step will be to evaluate the efficacy of these molecules within *in vivo* murine preclinical models, which will be crucial for further supporting the evidence base for onward translation.

**Reviewer #3 (Public review):**

In this work, the authors wanted to evaluate repurposed small molecule inhibitors for the treatment of envenomation by snakes of the Bothrops genus; one of the most medically relevant in the Americas. I believe the objectives of the research were clearly achieved, and compelling evidence for the ability of these molecules to neutralize enzymatic and toxic activities of metalloproteinases and phospholipases in all the tested venoms is provided. Furthermore, the work highlights the limited efficacy of the tested serine protease inhibitor, suggesting a need for drug discovery campaigns to address toxicity caused by this protein family. The methods are well designed and performed, and the use of both *in vitro* and *in vivo* methodologies makes this a thorough and robust work.

These results are extremely relevant, since they take us one step further to a potential orally administered snakebite treatment. The existence of such a treatment could improve the outcomes for thousands of snakebite victims worldwide. I have a few comments and questions that I hope will be useful to the authors:

We thank the author for their high regard for the purpose and execution of this work. Their insight in relation to questions are supportive for an improved manuscript and discussion points for the field.

During the introduction, the authors mention that small-molecule inhibitors can neutralize the localized tissue damage via cytotoxicity of some venoms, and cite PLA2s, SVMPs and/or cytotoxic 3FTxs as the main causing agents of this pathology. I am not aware of any direct effect described by small molecule inhibitors on cytotoxic 3FTxs alone. Has this been observed at all? Or is it more likely that the small molecule inhibitors act on the enzymatic toxins only, preventing synergistic effects with 3FTxs?

We apologise for this error on our behalf. While inhibitory molecules have been described for cytotoxic 3FTxs, these are not small molecules as alluded to in the previous version of the manuscript. We have amended this text in our revised manuscript.

I think it would be relevant to address the effects of non-enzymatic PLA2s, such as myotoxin II, which have been described in detail within Bothrops venoms. I believe there is some evidence of Varespladib also having a neutralizing effect on the myotoxicity caused by these non-enzymatic PLA2s. I suggest adding a comment about the contribution of these toxins in the discussion or in the section where PLA2 activity of the venoms is compared. In my opinion, right now it seems like these were overlooked.

We thank the reviewer for highlighting this point. We agree that this is highly relevant and would benefit from discussion in the revised manuscript given the nature of our assays and the non-enzymatic mechanism of action of certain Bothrops PLA_2_s. We have added this to the discussion.

Regarding Marimastat and the other MP inhibitors, are there any studies showing that they don't have an effect on endogenous MPs? I understand they have been approved for human use before, but is there any indication that they would not have an effect at the doses that would be required to treat envenomation?

Most matrix metalloproteinases inhibitors will act on endogenous MPs to at least some extent (variable potency on different MMPs). Marimastat has demonstrated activity against endogenous metalloproteinases, including MMP1, which was hypothesised to cause severe joint pain when used chronically (i.e. frequent dosing over many weeks) for indications such as cancer, though this effect was reversible within 8 weeks of cessation of drug administration (Wojtowicz-Praga, 1998). Thus long-term use of matrix metalloproteinases inhibitors can cause safety concerns. However, the anticipated duration of dosing for snakebite, which is an acute life-threatening condition, is a few days. It is therefore unlikely that prior safety concerns observed following chronic dosing in cancer studies would apply to its potential use as a snakebite field therapy.

Regarding the quenched fluorescence substrate used for enzymatic activity. Is there a possibility that some of the SVMPs would not act on this substrate, and therefore their activity or neutralization is not observed? Would it be relevant to test other substrates, such as gelatin, collagen, or even specific clotting factors?

It has been observed that certain SVMPs (specifically several PI SVMPs) are not active against this ES010 substrate *in vitro*. The substrate used in the *in vitro* SVMP assay is reported by the manufacturer as a substrate for a wide range of MMPs which target the extracellular matrix components mentioned by the reviewer, i.e. collagenases and gelatinases as well as matrilysins, stromelysins and elastate. This *in vitro* assay combined with the coagulation assays are complementary in covering the main targets of SVMPs (ECM and clotting cascade), prior to haemorrhagic assessment in the egg model. Thus, we are confident that activity for the broad range of SVMP isoforms will be captured through the screening pipeline we have developed.

Finally, could the authors comment or provide some bibliography regarding the translatability of the chicken embryo model in the context of envenomation?

Our current model is based on an earlier egg embryo model (Sells et al. 1997, Sells et al. 1998 and Sells et al. 2000) which described good correlations (p<0.01) with the standard WHO murine preclinical envenoming model. These studies have assessed correlations for minimal haemorrhagic doses (MHDs), LD50s and ED50s in both models for a selection of viper venoms. As chicken embryos at day 6 of development have incomplete neural arcs, the model is not well suited for assessing neurotoxic effects, but can be effectively used for addressing venom-induced haemorrhage and lethality and for testing therapeutics. In addition, a more recent study (Yusuf et al. 2023) reported almost identical LD50s for the venom of Bitis arietans between the two *in vivo* approaches. The model is also being pursued as a preclinical testing model by an antivenom manufacturer with the focus of reducing the use of rodents in batch release testing (Verity et al. 2021). We will provide further clarification on the rationale for using the egg model, including the supportive references outlined above, in the revised manuscript.

**Recommendations for the authors:**

**Reviewer #2 (Recommendations for the authors):**

The manuscript provides a useful comparative dataset across multiple Bothrops venoms and supports SVMP inhibition as a broadly effective lever in the authors in-vitro work. However, the strength of the 'pan-Bothrops' and translational claims is currently limited by insufficient characterization of the exact venom samples tested and by experimental designs that fall in clinically realistic rescue.

Major comments:

(1) The venoms used in this study are historical batches and are not formally characterized beyond SDS-PAGE and literature summaries, despite well-known intra- and inter-population venom variability; this weakens the generalization of the conclusions.

To address this comment, we have increased clarity on our venom sources being historic, Due to the historic source locality is not available beyond country of origin, with the exception of *B. lanceolatus* which is endemic to Martinique. Figure 1 also makes clear that we agree with the reviewer that the variation is high within Bothrops species. We discuss this variation on the limitations in our sampling for making broad conclusions throughout the first paragraph of the discussion, with the final sentence stating Future proteomic characterisations of the specific venom samples used in this study, which were all sourced from a historical collection (except for *B. lanceolatus*), would be informative in this regard. Although venom composition of our samples has not been characterised, the focus of the manuscript is the characterisation of the whole venom functional activity through a wide ranging screening pipeline, and the generalisation of our findings is supported by the diversity of the venom samples (i.e. several species) despite them not being characterised (which is not critical for the focus of the study).

(2) On a technical comment, the venom inhibition assays appear to rely on drug-first or preincubation conditions, which can easily overestimate efficacy compared with real snakebite envenomation, where toxins distribute and engage targets rapidly. Here, a translational gap is the clinical feasibility of the 'repurposed' inhibitors, as it is unclear whether the drugs central to the conclusions (especially marimastat, prinomastat and varespladib) are realistically available or stocked in hospitals or could be deployed in regions where Bothrops envenoming occurs. I think that the manuscript should clearly distinguish this from candidates with a plausible access and delivery pathway.

Our work in this manuscript includes standard practice of pre-incubation between drug and venom for all *in vitro* studies, and sequential (i.e. not co-incubation) administration in the egg model. None of our methods administer drug-first. Throughout the methods and figure legends we have made these distinctions clearer. Use of a ‘rescue’ approach in the *in vitro* assays is not feasible due to the rapid destruction of the substrates used for assay readouts. The clearest rationale for the use of rescue models relates to their power within *in vivo* preclinical models (i.e. murine envenoming models), which would be the next step for this research programme.

While the evidence presented in this manuscript is promising, there is much work still to do before such molecules are ready for deployment for treating snakebite, inclusive of the requirement to complete clinical trials, cost-benefit analysis and policy change and manufacturing/distribution feasibility assessments. Ultimately, this manuscript supports the growing evidence of the promising utility of marimastat and varespladib, and extends this evidence to neotropical snake venoms in a comparative manner. The next step will be to evaluate the efficacy of these molecules within rescue *in vivo* murine preclinical models, which will be crucial for further supporting the evidence base for onward translation. To further support this point we have included an additional section to the manuscript discussing the current preclinical and clinical progression of prinomastat and marimastat, which also incorporates the public comment on selection of marimastat over prinomastat.

(3) In my opinion, the Nafamostat results and discussion need reframing, given weak SVSP inhibition and intrinsic anticoagulant behavior at 5 µM. Excluding it from certain analyses undermines interpretability, and it may be more appropriate to include it throughout as an explicit negative control condition (showing its baseline anticoagulant effect) rather than omitting it.

Although we understand the reviewers opinion here, we disagree and believe that including nafomastat as a ‘negative control’ may present a negative reflection on the benefit that an efficacious serine protease inhibitor could provide. Furthermore, as the intrinsic anticoagulant effect of nafamostat cannot be de-coupled from direct SVSP toxin inhibition we were unable to interpret the activity which undermines the results. This can be seen in Figure 3b, which demonstrates that a false positive result would occur. For the serine protease assay, we do clearly discuss the lack of efficacy and justification of why EC_50_ testing wasn’t appropriate within the guidance of our screening protocols.

In the manuscript we have now further justified our approach in relation to the limitations of nafamostat as a snake venom serine protease (SVSP) inhibitor candidate due its low efficacy and off target effects. We highlight the need for the community to identify other serine protease inhibitors that might have utility for snakebite.

(4) The data presentation needs consistent statistical analyses (currently absent for multiple key figures, including Figures 2, 3, 4, 6 and 7) and a clearer explanation for the dose of venom and drugs you choose. For example, Figure 3 relies on a fixed 5 µM drug concentration and very different venom amounts (50-100-250 ng), but it is not discussed whether such exposures are achievable in vivo, or how these concentrations map onto expected pharmacokinetics in patients. Likewise, Bothrops venoms can contain both pro- and anticoagulant activities, so the authors should justify how their framework accounts for anticoagulant components and why the observed plasma phenotypes are interpreted as they are

In relation to the reviewers comment on the need for consistent analysis we thank the reviewer for flagging this and have now included these in figures 3, 4, 6 and 7. However, Figure 2 is presented to display the variation between all the venoms and ultimately used to select the most relevant doses for the latter inhibition experiments, therefore statistical analysis is not relevant for this figure. The updated statistical analysis now includes the following, which has been included in the relevant figure legends and results sections;

Figure 3 - Bars indicate significant results (p = <0.05) identified through one-way ANOVA with Dunnett’s multiple comparisons test to the DMSO control

Figure 4 - two-way ANOVA with Šídák's multiple comparisons test of each venom control compared to the matched venom treated with inhibitor

Figure 6 – the CT and MCF data were analysed independently using one-way ANOVA with Tukey’s multiple comparisons test

Figure 7 - Log-rank test (Mantel-Cox) with Holm- Šídák's multiple comparisons test against treatment vs venom-only control

We have ensured that all figure legends clearly indicate the venom and drug dose to aid the clarity which the reviewer requested.

The comment Figure 3 relies on a fixed 5 µM drug concentration and very different venom amounts (50-100-250 ng), but it is not discussed whether such exposures are achievable *in vivo*, or how these concentrations map onto expected pharmacokinetics in patients. is an understandable query however, *in vitro* assessment such as those carried out in this manuscript are not designed to directly inform pharmacokinetic/pharmacodynmanic interpretations, largely because they do not replicate real world envenoming (i.e. preincubation would not occur between a venom and treatment). This is why, as stated, follow on preclinical and clinical assessments are needed for onward progression of these inhibitors to inform dosing regimens that might achieve the necessary exposures required for *in vivo* venom neutralisation. That being said, PK/PD work has been initiated within Phase I trials, for example with DMPS Abouyannis et al. 2025 demonstrated a plasma exposure of >10 µg/mL for single doses of 1,200 mg and higher. This is equivalent to 80 µM, which although is lower than the EC_50_ for some venoms in the clotting assay (Figure 3J), the venom dose (50 to 250 ng/ 50 µL, i.e. 1,000 to 5,000 ng/µL) is estimated to be >1000 times higher than a natural envenoming by Bothrops atrox at less than 1 ng/mL in serum (https://doi.org/10.1016/j.toxicon.2022.09.010). These extrapolations therefore indicate that the doses selected in our studies would have human clinical relevance.

Finally, in terms of anticoagulant venom effects - these would be observed in our experimental approach either as reduced kinetic responses in the plasma clotting assay (as observed with nafamostat in Figure 3B) or as a prolonged clotting time in the thromboelastography assay (Figure 6). As stated in the results section Comparison of coagulation profiles, all of the venoms tested presented with a procoagulant effect. If underlying anticoagulant activity from PLA_2_ toxins was to arise after inhibition of the procoagulant toxins (i.e. SVMPs by marimastat), as has been seen for certain other snake venoms previously, this would result in a percentage inhibition far greater than 100% in the plasma assay (Figure 3C to I) or as a prolonged clotting time in the thromboelastography assay. These described anticoagulant profiles were not observed with any venom tested in this study.

(5) Finally, the in vivo evidence is limited to a chicken embryo model. To support your hypothesis, a conventional mouse model with delayed post-envenomation dosing (24-36 h monitoring) is needed to address both safety/toxicity and post-exposure efficacy, and to define a realistic therapeutic window, especially because venom toxins act very quickly and the timing of administration is central to the clinical utility of any small molecule approach.

We agree with the reviewer that the next important step for this research activity is utilising murine preclinical models to validate the *in vitro* and preliminary *in vivo* findings described in this manuscript. However, as stated above, this study provides the initial evidence base that the promising utility of marimastat, DMPS and varespladib as repurposed snakebite drugs extends to a range of neotropical viper venoms. Evaluating the safety, efficacy (both precincubation and rescue approaches) and PK/PD relationships to inform optimal dosing strategies of these molecules will be crucial next steps for the field. However, these activities are far from trivial and will take several years of additional research, and therefore fall outside the scope of this initial manuscript.

To address the concern related to the evidence is limited to a chicken embryo model, we have included additional sentences to discuss the wider use of the egg model within snakebite research and related translation to murine studies.

Minor comments:

(1) Figure 2D: How do you discuss the fact that "no venom" has SVSP activity?

The data for all *in vitro* assays in Figure 2 is presented as AUC from the raw data (absorbance or fluorescence), for consistency across assay. Therefore, all assays (B to D) have background signal in the absence of venom. The SVSP assay has a greater background signal.

(2) For better understanding, I would suggest adding a dedicated column in Figure 4A with Nafamostat SVSP data reported as "N/D" where applicable.

As stated in the results, due to the weak inhibitory activity EC_50_ assessment was not justified, therefore adding this column would be redundant.

(3) The introduction is too long relative to the experimental content and would benefit from tightening to sharpen the motivation and unmet need.

We thank the reviewer for their opinion and we have reviewed the introductory section again. While we made minor edits throughout, we decided not to make substantial modifications to it.

**Reviewer #3 (Recommendations for the authors):**

I only have some minor comments:

(1) In line 100, the word "that" is repeated.

We thank the reviewer for spotting this error, which we have corrected.

(2) Line 433. I believe the word "compromising" should be substituted by "comprising" here.

We thank the reviewer for spotting this.

(3) Figure 1 and supplementary: Bothrops asper venom has been very thoroughly studied, and using only one study from Costa Rica might underestimate the venom variation within the species. I suggest looking at the following study: https://doi.org/10.1016/j.toxicon.2022.106983. Maybe it is not necessary to change anything, but worth looking into.

We appreciate the reviewer flagging this paper, it has been added to the manuscript (reference 48) and has provided additional data for Figure 1 and Supplementary table 1.

(4) Methods: Given the intraspecies variation described for some of these species, I believe it is relevant to add the locality of origin of the venoms, and not only the country. I, of course, understand this is often unknown for historical samples.

We have included the following sentence in the methods. Due to the historic nature of the venom samples, the source locality is not available beyond country of origin, with the exception of *B. lanceolatus* which is endemic to Martinique.

(5) Figure 3: It is not very accurate to show an SD when the sample number is 2. I suggest, when possible, showing the mean and the two data points in the plots. This also applies to other figures where n=2. Also, in Figure 3D, does Marimastat seem to have an anticoagulant effect, or is this just within normal variation?

We have removed the statement in the statistics paragraph of the methods Standard deviation (SD) for all kinetic reads and standard error for AUC is reported based on Prism v10 but kept the sentence. The sample sizes for HTS assays including the SVMP, PLA_2_ and coagulation experiment are the average of the means from independent assays (n >2 within each independent assay). We understand the reviewer’s opinion on limited meaning of SD as well as SE for Fig 3 A to I, therefore we have changed the error bars to range, as we think that displaying the individual points would result in a lack of visual and analytic clarity.

In relation to the query about marimastat anticoagulant effect in Fig 4D, as shown in 4B marimastat has no direct anticoagulant effect. The >100% inhibition for marimastat is likely to be normal variation as this is a biological assay which has high variability. However, it could also be that the strong inhibition of the SVMPs in *B. asper* along with limited SVSP activity has unmasked an anticoagulant effect of the remaining PLA_2_ toxin which has high activity in this venom. That being said, as *B. asper* has a similar profile, we would have expected to see a similar profile in *B. atrox* in both the plasma and TEG assays. Therefore, assay variation seems the most likely reason for this observation.
